# Critical behaviours of contact near phase transitions

**DOI:** 10.1038/ncomms6140

**Published:** 2014-10-27

**Authors:** Y.-Y. Chen, Y.-Z. Jiang, X.-W. Guan, Qi Zhou

**Affiliations:** 1 State Key Laboratory of Magnetic Resonance and Atomic and Molecular Physics, Wuhan Institute of Physics and Mathematics, Chinese Academy of Sciences, Wuhan 430071, China; 2 Center for Cold Atom Physics, Chinese Academy of Sciences, Wuhan 430071, China; 3 Department of Theoretical Physics, Research School of Physics and Engineering, Australian National University, Canberra, Australian Capital Territory 0200, Australia; 4 Department of Physics, The Chinese University of Hong Kong, Shatin, New Territories, Hong Kong

## Abstract

A central quantity of importance for ultracold atoms is contact, which measures two-body correlations at short distances in dilute systems. It appears in universal relations among thermodynamic quantities, such as large momentum tails, energy and dynamic structure factors, through the renowned Tan relations. However, a conceptual question remains open as to whether or not contact can signify phase transitions that are insensitive to short-range physics. Here we show that, near a continuous classical or quantum phase transition, contact exhibits a variety of critical behaviours, including scaling laws and critical exponents that are uniquely determined by the universality class of the phase transition, and a constant contact per particle. We also use a prototypical exactly solvable model to demonstrate these critical behaviours in one-dimensional strongly interacting fermions. Our work establishes an intrinsic connection between the universality of dilute many-body systems and universal critical phenomena near a phase transition.

## Abstract (web summary)

Contact parameterises two-body correlations at short distances in dilute systems like ultracold atomic gases. Using a fundamental thermodynamic relation, Chen *et al.* study the contact near a continuous classical or quantum phase transition and find that it displays a number of critical behaviours.

## Graphical abstract

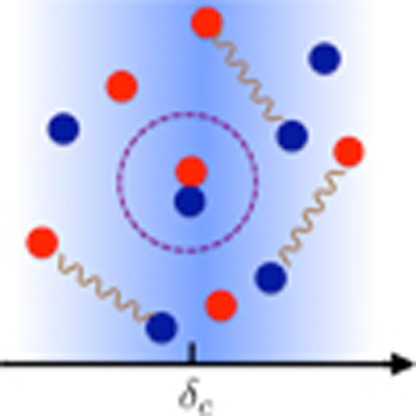

The notion of contact 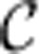 strikingly captures the universality of ultracold atoms. As revealed by the Tan relations^1^,^2^,^3^ and their expressions in other forms^4^,^5^,^6^,^7^, regardless of the choice of microscopic parameters, a wide range of quantities in dilute systems is governed by 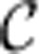 that characterizes the probability that two particles may be separated by a short distance less than *d*. For instance, when *d* approaches zero, the two-body correlation function of two-component fermions in three dimensions follows 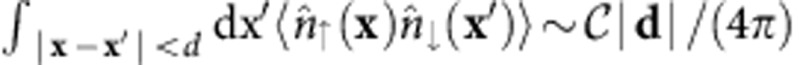, where 
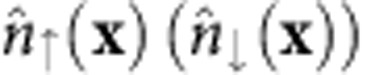
 is the density operator at position **x** for spin-up(down) particles. While the definition of contact is apparently independent of the many-body phase the system is exhibiting, there is much interest in exploring the behaviour of contact near a phase transition^8^,^9^,^10^,^11^,^12^,^13^,^14^,^15^,^16^,^17^. The success of such efforts will significantly deepen our understanding of the connection between short-ranged two-body correlations and phase transitions, which are generally believed to be disentangled from each other, since the latter is insensitive to the details of short-range physics.

Even though the contact of strongly interacting fermions remains finite in both the normal and the superfluid phase, experimental studies have provided evidence indicating that it gets enhanced near the superfluid transition temperature. However, owing to a lack of resolution, it is unclear whether contact exhibits any critical features near the transition point. On the theoretical side, it is extremely difficult to exactly calculate the contact of strongly interacting fermions near the transition temperature, and therefore certain approximations have to be made. Theoretical approaches based on different techniques lead to contradictory results^10^, ranging from a kink to a discontinuous jump of the contact near the transition point. Therefore, it is of fundamental importance to provide a concrete answer for the relation between contact and phase transitions.

In this work, our approach is to derive exact results on the behaviour of contact near a classical or quantum phase transition based on a fundamental thermodynamic relation that is free from any approximations. These results unambiguously show that contact must display critical behaviours near the transitions, and that the corresponding critical behaviours are uniquely determined by the universality class of the phase transition. We use a one-dimensional (1D) exactly solvable model of strongly interacting fermions exhibiting exotic quantum phase transitions to demonstrate these critical phenomena. Our exact result for contact is obtained from the Bethe ansatz for a 1D Fermi gas that provides a precise understanding of critical phenomena beyond the Tomonaga–Luttinger liquid (TLL) physics. Whereas our general results apply to all dimensions, this 1D example sheds light on the universal features of contact near a phase transition.

## Results

### Critical behaviours of contact in three dimensions

We consider the fundamental thermodynamic relation,


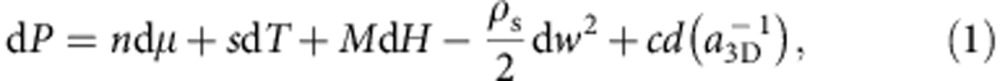


where *P* is the pressure, *n*, *s*, *M*, *ρ*_s_ and 
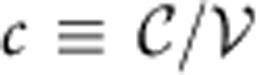
 are the densities of the particles, the entropy, the magnetization, the superfluid and the contact, respectively, 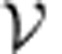 is the volume of the system, and *μ*, *T*, *H* and *a*_3*D*_ are the chemical potential, temperature, magnetic field and scattering length, respectively. Compared with the usual definition of contact, here the prefactor *ℏ*^2^/(4*πm*) (with *m* denoting the mass) has been absorbed into *c*. In this relation, *w*=*v*_s_−*v*_n_ is the difference between the velocity of the superfluid and normal components, which can be generated by slowly rotating the atomic cloud so that the critical velocity of the superfluid is not reached anywhere in the trap. Equation (1) has been used to measure thermodynamic quantities such as the pressure, the equation of state and density–density response function^18^,^19^,^20^,^21^,^22^.

Compared with the original Tan relation 
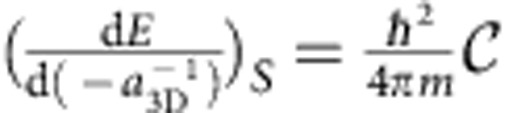
, where *E* and *S* are the total energy and entropy of the system, respectively, equation (1) has the advantage of allowing one to directly correlate the contact with phase transitions for both classical and quantum ones. First, as *c* is a partial derivative of the pressure, that is, 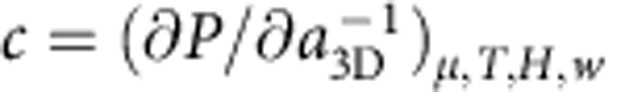, as are *n*, *s M* and *ρ*_s_, equation (1) tells one that contact near the critical point must exhibit critical behaviour determined by the universality class of the phase transition. In particular, the contact should vary continuously across a continuous phase transition. For instance, across the superfluid phase transition of strongly interacting fermions in three dimensions, *c* is continuous. Second, the Maxwell relations derived from equation (1) show that the derivatives of the contact with respect to *μ*, *T*, *H* and *w* exhibit critical behaviour. These Maxwell relations can be written as


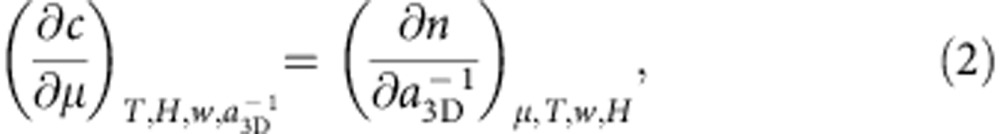



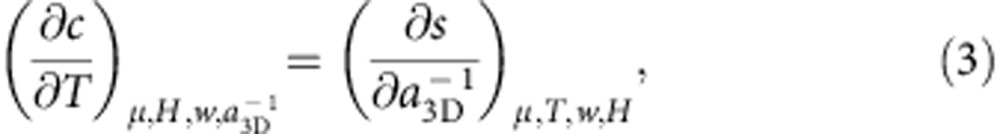



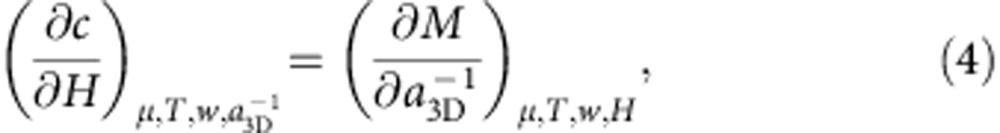



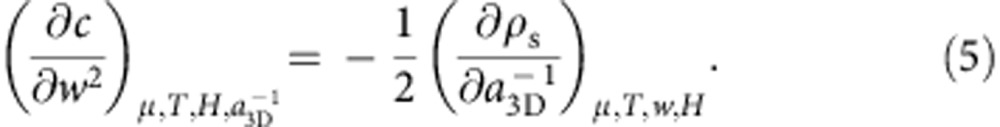


The exact relations (equations (2)–(5)) bring new physical insight into the correlations between the contact and other physical quantities, including the magnetization and superfluid density that characterizes magnetic and transport properties, respectively, which have not been explored in the literature. Among these exact relations, equation (5) is of particular interest. It directly correlates the contact with *ρ*_s_ characterizing superfluid phase transitions. Despite *c* being finite in both the normal and superfluid phases, there is a difference. Equation (5) shows that in the normal phase, 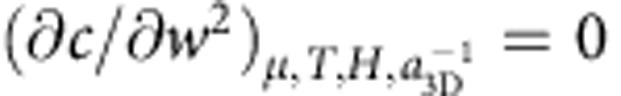, and the contact remains unchanged after a slow rotation is turned on, since *ρ*_s_≡0. In the superfluid phase, *ρ*_s_ in general depends on the scattering length, and therefore 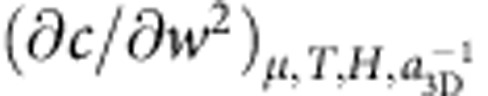 is finite. In particular, in a stationary system with *w*=0, *ρ*_s_ follows the standard scaling law near the transition point, 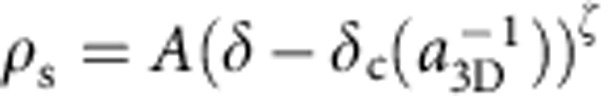, where the tuning parameter *δ* can be *T* and *μ* (or *H*) for classical and quantum phase transitions, respectively. Here, *A* is independent of *δ*, and *ζ* is the corresponding critical exponent. One then obtains the scaling law for 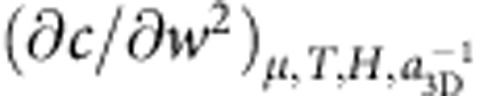 near the transition point,


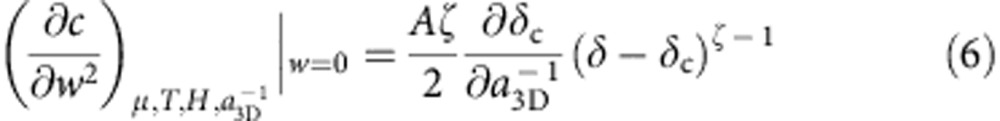


Equation (6) shows that the exponent of 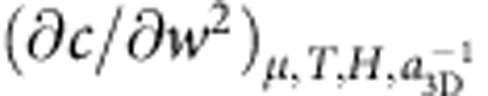 is entirely determined by the universality class of the superfluid phase transition. The above properties of the contact can be easily tested in experiments on trapped atoms, where the superfluid and normal phases are distributed in different regions of the trap. With the high-resolution images available in current experiments, the local contact density *c* can be extracted as a function of *μ* using 
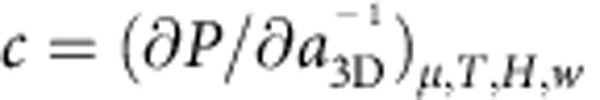
. One then could examine the distinct responses of *c* to rotation, 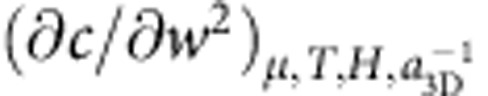, in both the superfluid and normal phases.

Equations (2)–(4) can also be experimentally tested. Near the phase transition point, the scaling law in a system with *w*=0 for a quantity *O* takes the form 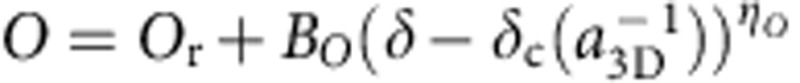, where *O*=*n*, *M* or *s*, *O*_r_ is the regular part of *O* and *η*_*O*_ is the corresponding critical exponent. One then obtains:


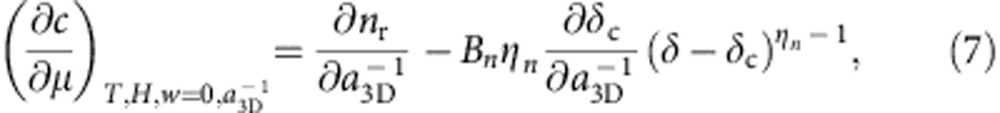



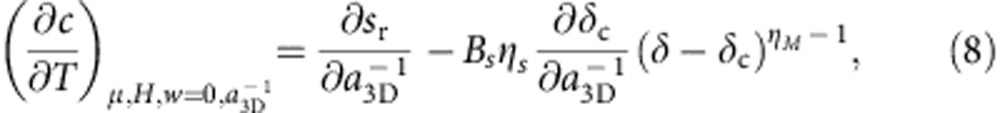



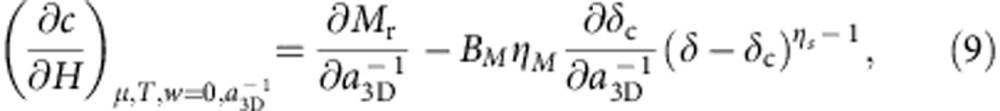


where the subscripts on the derivatives of *O*_r_ have been suppressed. The above differential forms also indicate scaling laws for *c*. For instance, if one chooses *δ*=*μ*, then from equation (7) one obtains 
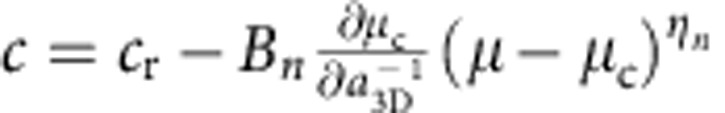
, where 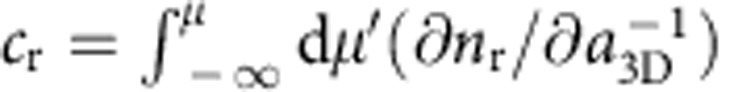. Note that the dependences of *c* and *n* on *μ* have the same exponent, so that one concludes that


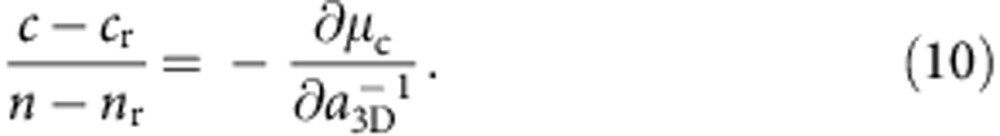


In particular, if *c*_r_=*n*_r_=0, one sees that the contact per particle in the critical region becomes a constant that is entirely determined by 
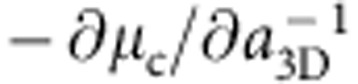
. As the non-uniform distribution of trapped atoms allows experimentalists to trace the dependence of the contact on the chemical potential^18^, equations (7) and (10) can be directly tested in experiments.

### Contact in an exactly solvable 1D Fermi gas

Whereas the above discussion applies to all ultracold atomic systems, it is particularly interesting to use an exactly solvable model to demonstrate some of the critical behaviour of contact. Here, we consider equations (7) and (10), since they can be implemented in experiments easily without a rotation. In one dimension, equation (1) becomes


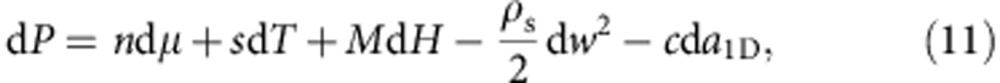


where *a*_1D_ is the 1D scattering length and *c* differs from the ordinary definition by a trivial prefactor *ℏ*^2^/(2*m*). Each of the equations (1)–(10) has a direct analogue in one dimension that is obtained by the simple replacement 
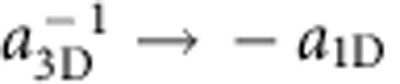
. We study a 1D Fermi gas with *δ*-function interactions, described by the Yang–Gaudin Hamiltonian^23^,^24^,^25^


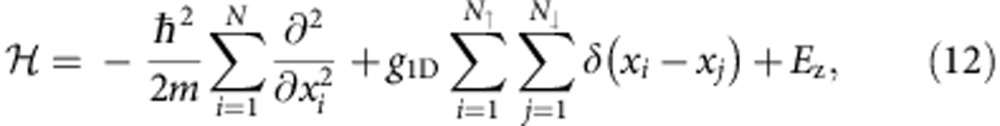


where 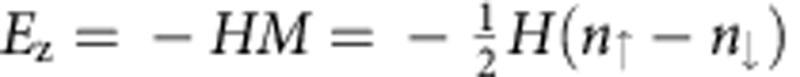 is the Zeeman energy induced by a magnetic field *H*, 
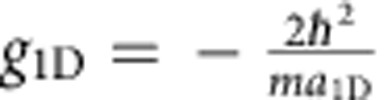
 characterizes the interaction strength determined by the effective 1D scattering length 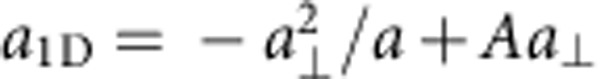
26, *a*_⊥_ is the transverse oscillation length and *A*≈1.0326. We introduce the polarization *P*=(*n*_↑_−*n*_↓_)/*n*, and define a dimensionless interaction parameter γ=*mg*_1D_/(*nħ*^2^)=−2(*na*_1D_)^−1^ for our analysis, choosing natural units 2*m*=*ℏ*=*k*_B_=1.

The model described by equation (12) has been solved using the Bethe ansatz^23^,^24^ and has had a tremendous impact in statistical mechanics. The experimental developments in studying 1D fermions^27^,^28^,^29^,^30^,^31^ have inspired significant interest in relating theoretical results to experimental observables^25^,^32^. It was found^33^,^34^,^35^ that although the thermodynamic Bethe ansatz (TBA) equations involve non-trivial collective behaviour of the particles, that is, the motion of one particle depends on all others, the total effect of the complex behaviours of all the individual particles leads to qualitatively new forms of simplicity in many-body phenomena^36^,^37^.

Contact of the ground state, in the extremely polarized limit with a single spin-down atom, has been studied in refs 38, 39. However, reaching the goal of finding critical behaviours of contact requires a theoretical framework, beyond mean-field theory, capable of analytically deriving the thermodynamic properties of such gases at finite temperatures (Supplementary Note 1). This has been a fundamental challenge in theoretical physics owing to the strong interaction between the atoms. Here we compute the contact by numerically solving the TBA equations and obtaining analytic expressions in the physical regime 
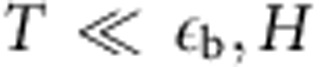
 and 
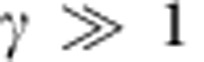
, where 
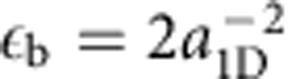
 is the binding energy of the pairs, and explore its behaviour near the phase transition. Even though there is no finite-temperature phase transition in one dimension, there does exist a universal finite-temperature crossover that remarkably separates the low-energy critical TLL with relativistic dispersion from the collective matter of free Fermi criticality with non-relativistic dispersion. Moreover, quantum phase transitions between two of the following phases in this model, the vacuum phase (V), the fully paired phase (P), the fully polarized phase (F) and the partially polarized phase (PP)^36^,^37^, provide a precise description of the critical behaviours exhibited by contact in many-body systems.

**Figure 1:**
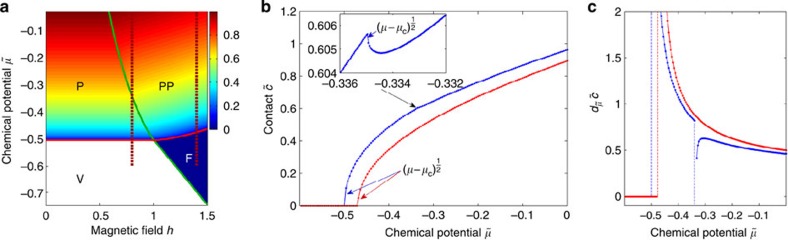
Contact of 1D two-component fermions with zero range interaction at zero temperature. (**a**) A contour plot of contact as a function of the dimensionless chemical potential 
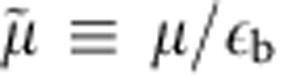
 and magnetic field 
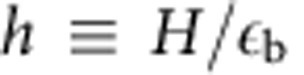
, where 
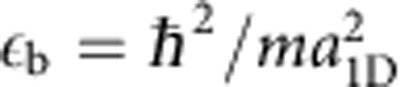
 is the binding energy determined by the 1D scattering length *a*_1D_. The notations V, P, F and PP stand for vacuum, fully paired, fully polarized and partially polarized phase, respectively. Red and green curves represent the phase boundaries obtained from thermodynamic Bethe ansatz equations. Vertical dashed lines correspond to two cuts at fixed *h*=0.8 and 1.4. (**b**) Contact is continuous across the quantum critical points. For the transition V–P and F–PP, the dimensionless contact 
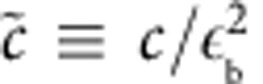
 continuously increases from zero as 
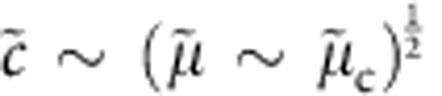
. For the transition P–PP, contact is finite on both sides of the transition point and a kink exists as 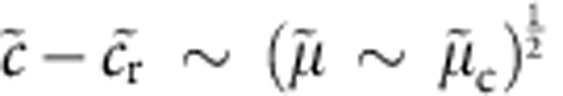, indicating the discontinuity of the derivative of contact. (**c**) The derivative of contact with respect to 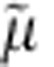 becomes divergent as 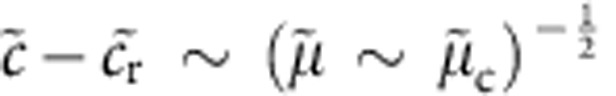 in this 1D system at all the transitions V–P, P–PP and F–PP for fixed values of *h*=0.8 (blue line) and *h*=1.4 (red line).

The phase diagram Fig. 1 shows numerical results for the dimensionless contact density 
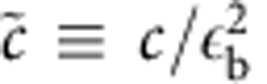
 at zero temperature as a function of the dimensionless chemical potential 
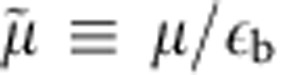
 and magnetic field *h*≡*H*/ε_b_, where *c* is obtained from the TBA equations (Supplementary Note 1) through *c*=−(*∂P*/*∂a*_1D_)_*μ*,*H*,*T*_, and *w* has been set to be zero. Here we have chosen ε_b_ as the energy scale. Alternatively, one may choose the Fermi energy, *E*_F_, which will not change the later discussion and results. Since we have chosen natural units by setting *ℏ* and 2*m* to be 1, *c* has the same dimension as 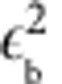 so that 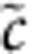 as defined is dimensionless. Across the transition from V to P, the regular part 
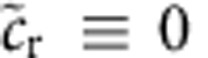
, since 
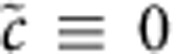
 in V, and past the critical point 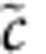 continuously increases from zero as 
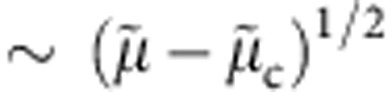
. Correspondingly, in P, 
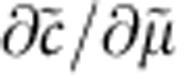
 diverges as 
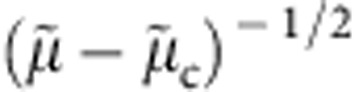
 at this transition point. The aforementioned scaling laws for 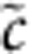 and 
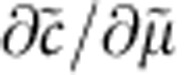
 are derived directly from the zero temperature scaling law for density near this critical point, 
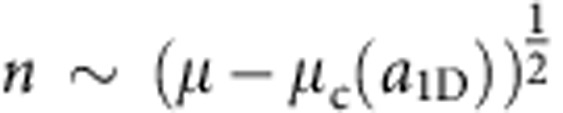
. By taking the derivative of *n* with respect to *a*_1D_, one sees that the critical exponents for 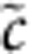 and 
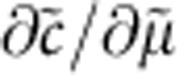
 are indeed 1/2 and −1/2, respectively. Near the other transition point from P to PP, *c* also changes continuously with a kink 
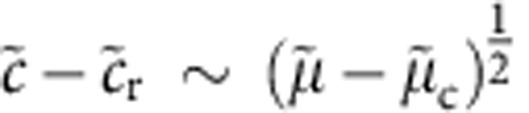
 and 
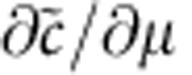
 has the same 
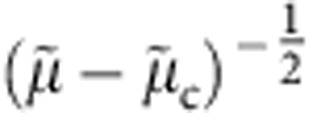
 divergence.

At finite temperatures, 
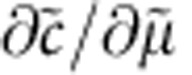
 no longer diverges (Supplementary Fig. 1). Nevertheless, critical phenomena exist for both 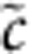 and 
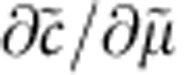
 in a region expanded to finite temperatures, as is typical for quantum criticality. We work out the analytic expressions for 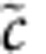 and derive the scaling form for 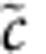 and its derivatives in the quantum critical region. For the physical regime, 
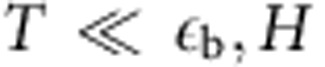
 and 
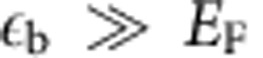
, 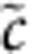 is given explicitly by


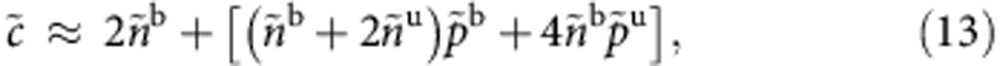


where


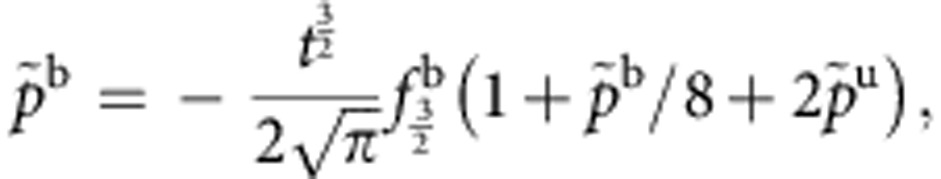



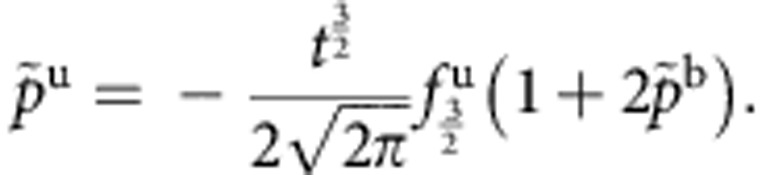


Here, we use the notation 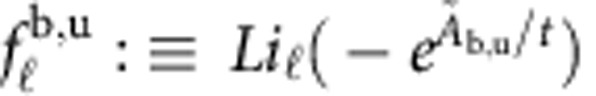, 
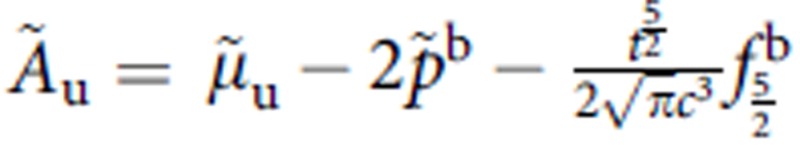
, 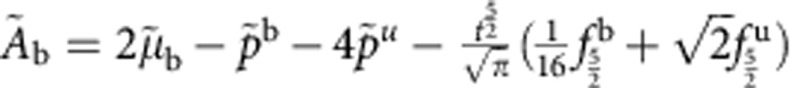, and 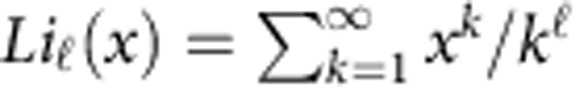 is the polylog function. We have defined *t*≡*T*/*ε*_b_, 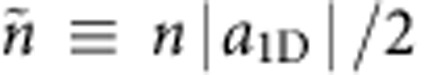, and 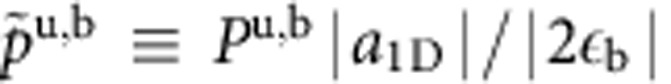, where the labels b and u indicate if a quantity describes a property of bound states or unpaired particles. The result (equation (13)) is valid for both the TLL phase and the critical region (Supplementary Fig. 2). Physically, 
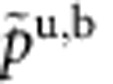
 and 
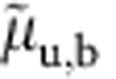
 represent the dimensionless pressure and chemical potential of unpaired fermions and bound pairs, respectively. Owing to the residual interaction between them, 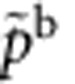 and 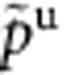 are correlated through the above coupled equations.

It is interesting to note that, apart from a small correction 
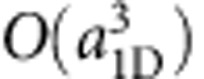
, the terms within the square brackets of (equation (13)) give the pressure of the interacting system after subtracting that of an ideal gas consisting of single fermionic atoms with mass *m* and composite atoms with mass 2*m*, namely


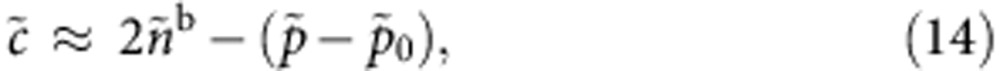


where up to the order of 
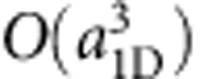
, the pressure is 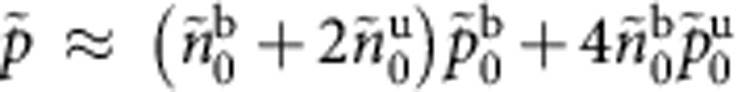. In these equations, 
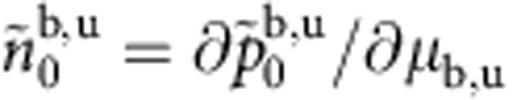
 and 
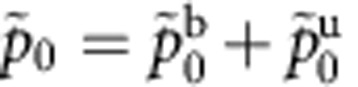
, with 
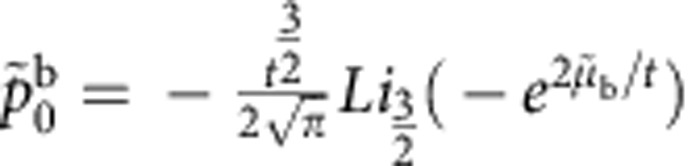
 and 
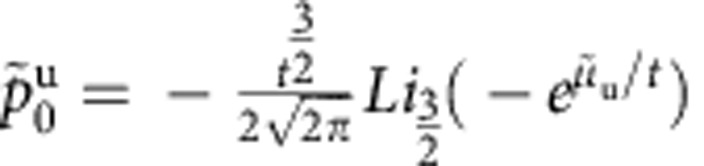
 (Supplementary Note 1). Physically, 
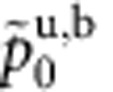
 represents the pressures of free unpaired fermions or bound pairs, as one sees clearly that their expressions are identical to those for non-interacting particles. The term in the parentheses of equation (14) reveals an important characteristic of contact in the strongly interacting region: it accounts for the interaction between bound pairs, and that between pairs and unpaired fermions, in addition to the contribution from each pair itself. The high-order corrections to contact from multibody interaction effects, that is, scattering involving three pairs, are relatively small in the strong-coupling regime. In this regard, the two-body interaction, including both pair–pair and pair–unparied fermions scattering, are important for determining the critical behaviours of contact in a strongly interacting Fermi gas. On the other hand, in order to capture proper thermal and quantum fluctuations in the quantum critical region, the universal scaling behaviour of the contact requests such marginal contributions from those higher order corrections (Supplementary Note 1).

**Figure 2:**
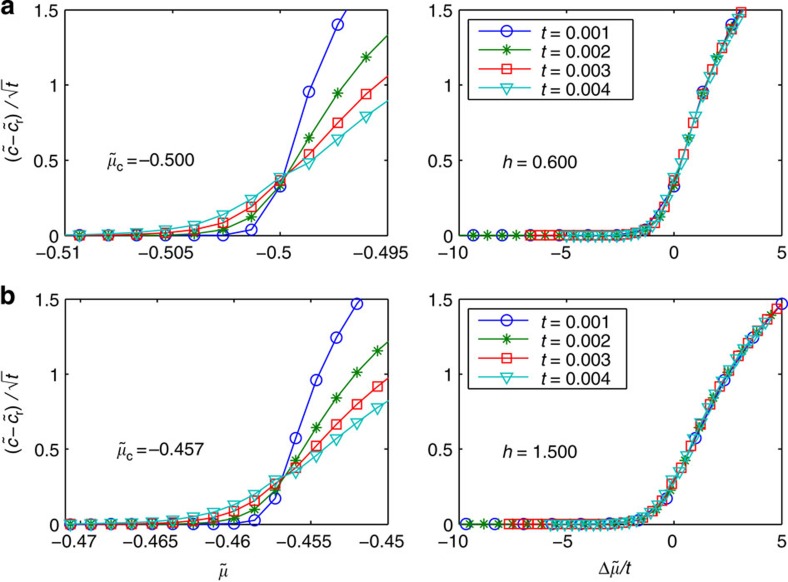
Scaling laws of contact determined by the universality class of the phase transition in the quantum critical region. The left panels show the temperature-scaled contact 
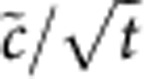
, where *t*=*T*/*ε*_b_ is the dimensionless temperature, as a function of the chemical potential near the phase boundaries V–P (**a**) and F–PP (**b**), respectively. Curves at different temperatures intersect at the quantum critical point as predicted by equation (15). The right panels show that the rescaled contact versus temperature-scaled chemical potential 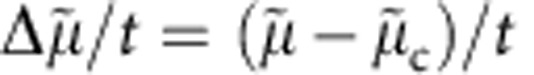 at different temperatures collapse into a single line, characteristic of critical behaviour in the quantum critical region. These data collapses confirm the critical dynamic exponent *z*=2 and correlation length exponent *ν*=1/2 in terms of the universal scaling in equation (15).

Using equation (13), we find the universal scaling form of *c* in the quantum critical region,


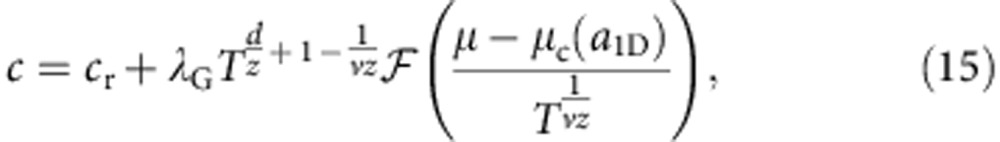


where *c*_r_ is a temperature-independent regular part, the constant *λ*_G_ depends on *μ*_c_, and 
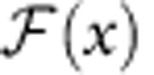
 is a dimensionless scaling function that can be determined by the TBA equations (Supplementary Note 1). The dynamic and correlation length exponents have been found to be *z*=2 and *ν*=1/2, see the data collapse after use of scaling law (equation (15)) in Fig. 2. From equation (15), we obtain:


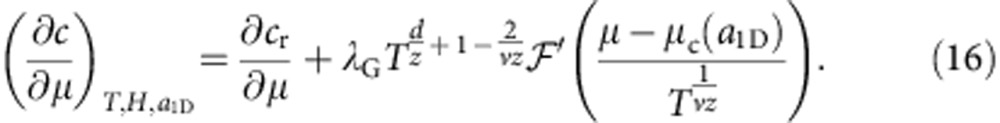


**Figure 3:**
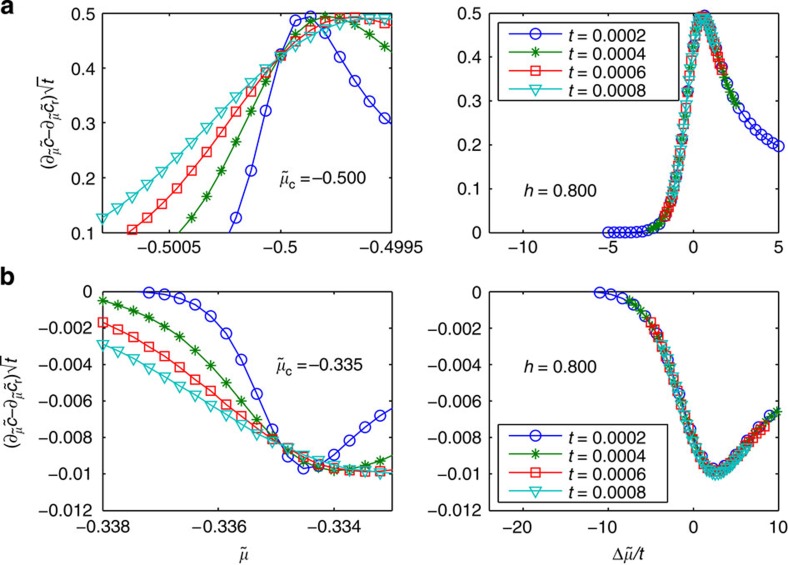
Scaling laws of the derivative of contact with respect to the chemical potential in the quantum critical region. The left panels show that different curves of 
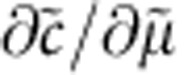
, where 
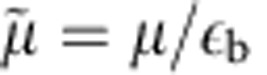
 is the dimensionless chemical potential, near the transition V–P (**a**) and P–PP (**b**) intersect at the quantum critical point. The right panels show the collapse of 
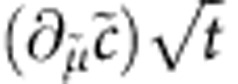
 versus the temperature-scaled chemical potential 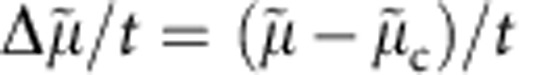 into a single curve. Results in both panels confirm the scaling laws predicted by equation (16). From the temperature-scaled contact at different temperatures, one reads off the the critical dynamic exponent *z*=2 and correlation length exponent *ν*=1/2.

Figure 3 shows the scaling behaviour of this derivative of contact. Similar results can be obtained if one chooses *H* as the tuning parameter (Supplementary Figs 3 and 4). Comparing equation (15) with the standard scaling form of the density in the quantum critical region, 
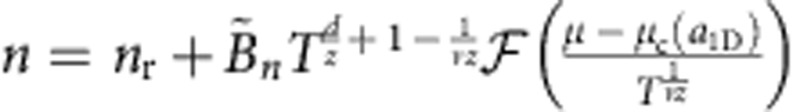
, we find that 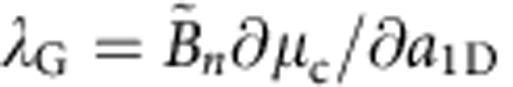 and


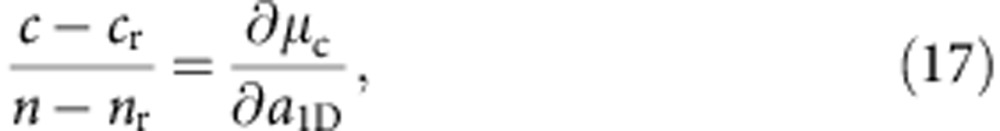


in analogy to equation (10) in three dimensions. For the phase transition V–P, 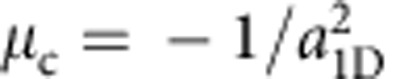 and 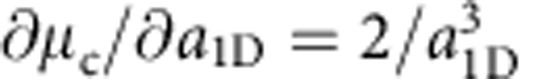, so that equation (17) can be rewritten as 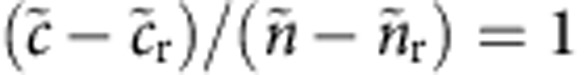. For other phase transitions, such as P–PP and F–PP, this ratio has different constant values. The scaling forms, equations (15) and (16), lead to the intersection of the scaled quantities 
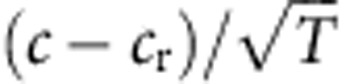
 and 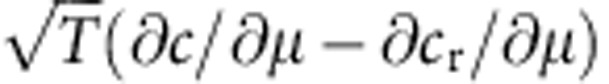 for different temperatures at *μ*_c_ in our system. If one further plots these quantities as functions of (*μ*−*μ*_c_)/*T*, different curves collapse to a single one. Such intersections and data collapses are characteristic for the quantum critical behaviour of contact. We have numerically confirmed the validity of these scaling forms for all interaction strengths.

**Figure 4:**
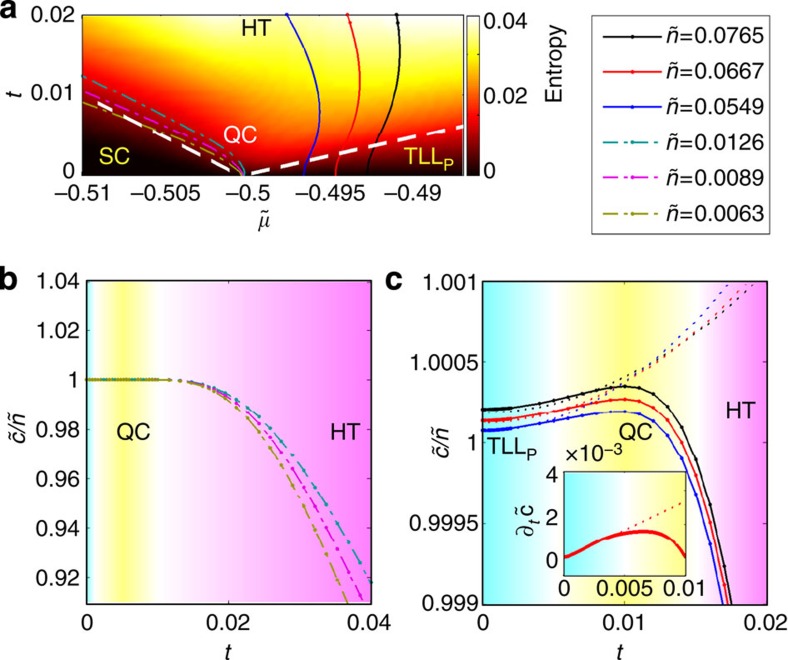
Contact per particle at finite temperatures. (**a**) Density plot of the entropy obtained from the numerical solution of thermodynamic Bethe ansatz (TBA) equations for highlighting different regions for the phase transition V–P on the 
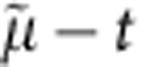
 plane. We denote by SC the semiclassical region with very low density. QC stands for the critical regime with non-relativistic dispersion, TLL_p_ is the Tomonaga–Luttinger liquid (TLL) of pairs with linear relativistic dispersion and HT stands for high temperature region where universal behaviour of thermodynamics are absent. Dashed white lines represent the crossover temperatures from QC to SC and TLL regions. (**b**) Contact per particle 
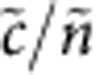
 versus the temperature at fixed values of low densities, where 
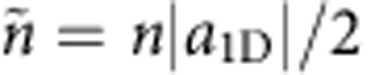
 is the dimensionless density. The flatness of 
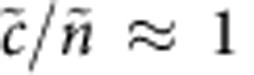
 confirms the constant contact per particle as shown in equation (15) in the QC region. (**c**) 
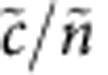
 at high densities. The solid lines show the numerical result derived from TBA equations. The deviations of the TLL result (dotted lines) from TBA results indicate the breakdown of the TLL_p_ phase at the crossover temperature T* from TLL_p_ to QC. T* here is consistent with the result obtained from the deviation of entropy from the linear temperature dependence of TLL. The inset shows 
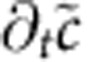
 versus temperature in which the deviation is more visible. A maximum of 
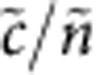
 demonstrates the enhancement of contact when quantum effects become important in the quantum degenerate region where 
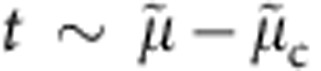
.

We now turn to the contact per particle in the quantum critical region. To highlight the quantum critical region and other ones in the 
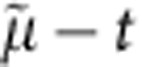
 plane, Fig. 4 shows a density plot of the entropy. The regions are separated by a crossover temperature T*, shown by the white dashed line in Fig. 4. The crossover from the quantum critical region to the TLL region, where the density is finite for 
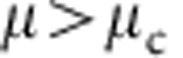
 at zero temperature, is obtained from the deviation of the entropy from the linear form of TLL^25^. On the other side of the transition point, the crossover temperature from the quantum critical region to the semiclassical region, where the density is exponentially small, is obtained by setting the thermal wavelength equal to the interparticle spacing. In Fig. 4a, three curves are shown for the rather small fixed values of the density 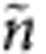 listed in Fig. 4b. One can see that a very large portion of the trajectory at such constant densities remains in the quantum critical region. As a result, 
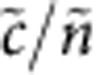
 becomes 1. In Fig. 4b, numerical results for the scaled contact per particle for these three densities are shown to satisfy 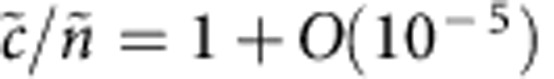 up to the temperature scale *t*=10^−2^, which corresponds to a ratio of the temperature to the chemical potential 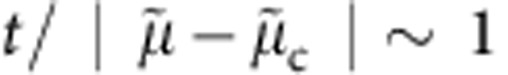. These results directly confirm equation (17).

At higher densities and with increasing temperature, the trajectory at constant density first enters the TLL region, quickly passes the quantum critical region and eventually enters the high temperature region with 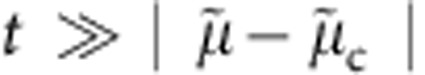, where the entropy density becomes large and the universal scaling laws of contact fail, as shown in Fig. 4a. Below T*, and in the TLL phase of the paired fermions, referred to as phase TLL_p_, the contact in the strong-coupling regime is given by


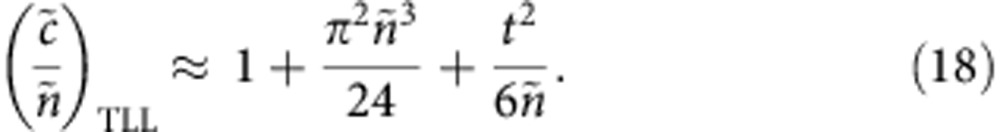


Figure 4c shows both the numerical results of 
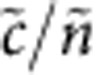
 at large densities and the result of the TLL theory based on equation (18). It is clear that the growth of 
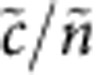
 at low temperatures is described well by equation (18). The deviation from the TLL result shows a breakdown of the TLL at crossover temperature T*. More interestingly, one sees that before 
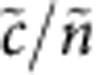
 eventually decreases at high temperatures, a maximum occurs around *t*≈0.01 (about 0.1–0.5 *T*_F_ for strong attractive regime^28^), which corresponds to a quantum degenerate temperature 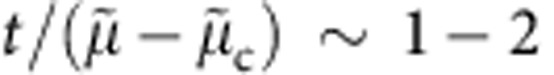. Such a maximum indicates that the contact per particle gets enlarged in the quantum degenerate region, similar to the possible enhancement of the contact near the transition temperature of three-dimensional (3D) fermions^10^.

## Discussion

Whereas the Tan relations have revealed how contact controls various thermodynamic quantities, it is in general difficult to make quantitative predictions as to how contact depends on the many-body physics of the system. Our results have shown that in the critical region near a phase transition point, contact and its derivatives are uniquely determined by the universality class of the phase transition. The exact thermodynamic relations shown in equations (1)–(5) lead to both new insights into fundamental physics and profound applications for connecting contact and macroscopic quantum phenomena. Whereas these relations are exact for any microscopic parameters, they are particularly useful in the critical region for establishing exact relations between the universal scaling behaviours of contact and those of other thermal, magnetic and transport quantities. In particular, we have proved that contact in one dimension not only provides an unambiguous determination of the TLL phases and but also identifies in a novel fashion the universality class of quantum critical interacting many-body systems.

Moreover, equations (1)–(5) can be used to ultimately settle the aforementioned controversy over the contact of the 3D unitary Fermi gas near the superfluid phase transition point. On the experimental side, our results suggest that high-resolution *in situ* images may be used to obtain precision measurements of the local pressure and contact as a function of temperature and other microscopic parameters, so that an average in the trap is not necessary. Such experiments will also be useful for exploring the size of the critical region, which is predicted to be of the order unity in the unitary limit^40^. On the theoretical side, whereas a number of approaches have obtained a continuous contact across the transition point, consistent with the prediction of our exact thermodynamic relations, one needs to examine whether the results produced by a theory indeed satisfy the exact thermodynamic relations in equations (1)–(5).

In this Article, we have focused on continuous phase transitions, where all physical quantities, including both superfluid density and contact, are continuous across the transition point. It is worth pointing out that a unique phase transition occurs in two-dimensional (2D) superfluids, where the superfluid density has a finite jump, and meanwhile other thermodynamic quantities remain continuous, at the Berezinskii–Kosterlitz–Thouless transition point. It would be interesting to explore whether contact could signify such a finite jump of superfluid density controlled by the deconfinement of topological excitations, that is, vortices in 2D superfluids.

Highly controllable ultracold atoms are ideal platforms for exploring both universality of dilute systems governed by contact and universal critical phenomena near a phase transition point in many-body systems. In particular, current experiments with ultracold atoms are capable of measuring the critical behaviours of contact in all dimensions. We hope that our work will stimulate more studies on the intrinsic connection between these two types of fundamental phenomena on universality in physics.

## Methods

### The model

For the attractive spin-1/2 Fermi gas at finite temperatures, the thermodynamics of the homogeneous system is described by two coupled Fermi gases of bound pairs and excess fermions in the charge sector and ferromagnetic spin–spin interaction in the spin sector, namely the TBA equations read^35^


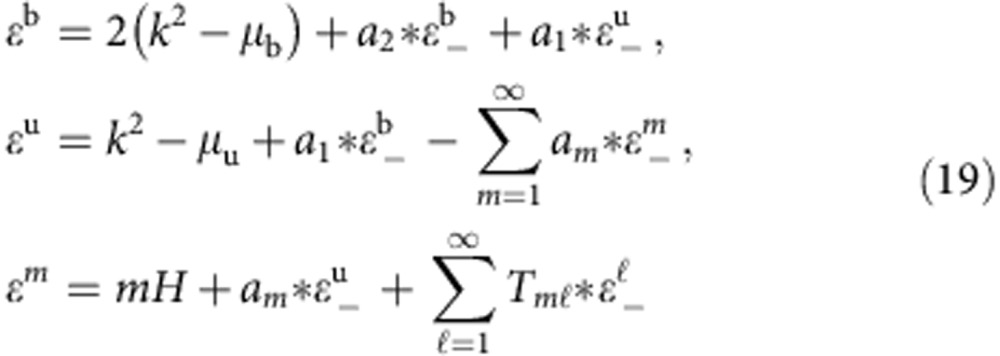


with *m*=1,…∞. In the above equations, * denotes the convolution integral, 
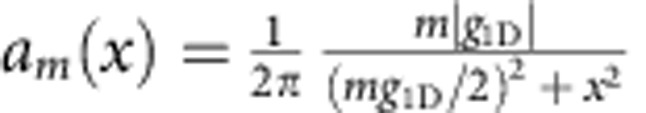
 and 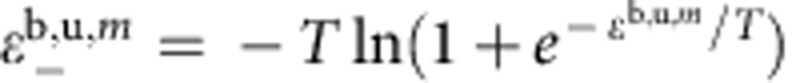. Here *ε*^b,u,*m*^ are the dressed energies for bound pairs, excess single fermions and *m*-strings of spin wave-bound states, respectively. These dressed energies account for excitation energies above Fermi surfaces. In the above equations the function 
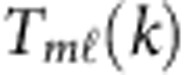
 is given by *T*_*nm*_ (*k*)=*A*_*nm*_ (*k*)−*δ*_*nm*_ *δ*(*k*) with *A*_*nm*_=*a*_|*n*−*m*|_+2*a*_(|*n*−*m*|+2)_+⋯+2*a*_(*n*+*m*−2)_+*a*_(*n*+*m*)_; see ref. 35.

The effective chemical potentials of unpaired fermions and pairs were defined by *μ*_u_=*μ*+*H*/2 and *μ*_b_=*μ*+*ε*_b_/2. The thermal potential per unit length *P*=*p*^u^+*p*^b^ is given in terms of the effective pressures 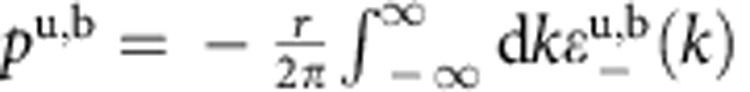 with *r*=1 and 2 for the unpaired fermions and bound pairs.

The strategy for working out scaling form of contact near the critical points is to, at first, perform analytical calculation of contact near different phase transition points in the physical regime 
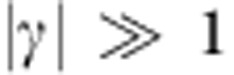
 and 
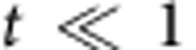
. Then we confirm the analytical result of the universal scaling forms by numerically solving the TBA equations of the model for all interacting strengths. To this end, we first present the analytical expression of the total pressure *P*=*p*^b^+*p*^u^ for the regime 
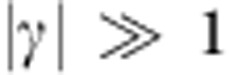
 and 
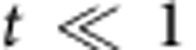
 (ref. 37).


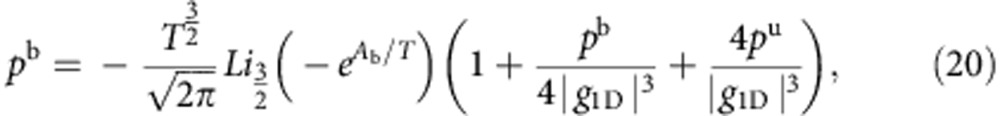



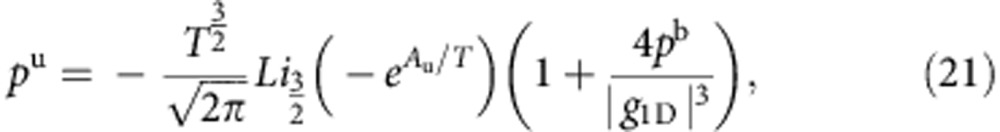


with the functions


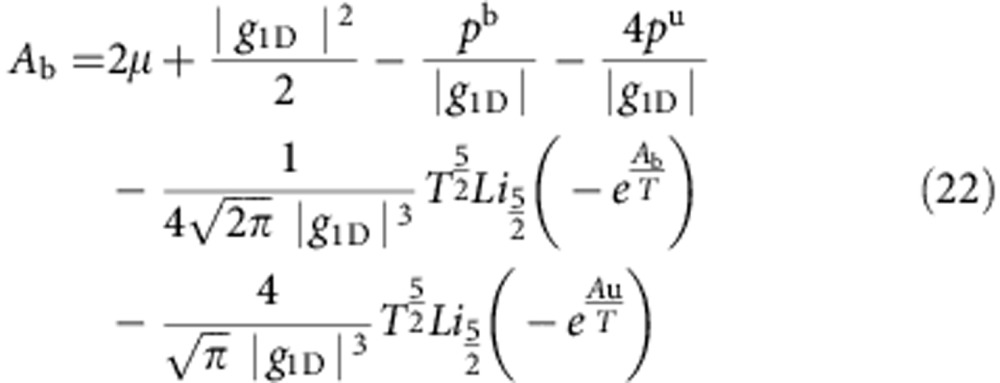



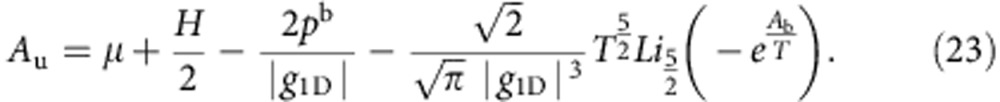


In this model, the *SU*(2) spin degree of freedom ferromagnetically couples to the unpaired Fermi sea. Thus, the spin wave contributions to the function *A*_u_ is negligible owing to an exponentially small contributions at low temperatures^37^. By iteration, these effective pressures of bound pairs and unpaired fermions *p*^b,u^ can be presented in close forms. Here a significant observation from equations (20) and (21) is that the pressure *P* can be written in term of a universal scaling form near the critical fields, that is,


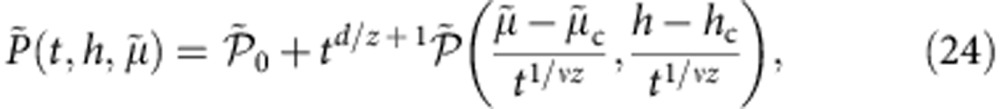


where the dimensionless pressure 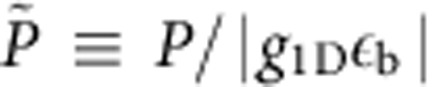, _0_ is the background pressure and is the dimensionless scaling function. The dimensionless critical chemical potential 
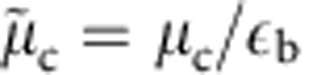
 and critical field *h*_c_=*H*_c_/*ε*_b_ depend on the interaction strength *g*_1D_. Therefore, contact would essentially possesses universal scaling form near each critical point.

### Numerical method

In principle, the TBA equation (19) in the paper present full thermodynamical properties of the model for all temperature regimes and interaction strength. Analytical result obtained above are useful for carrying out full thermodynamics of the model throughout all interaction regimes. In the present paper, the numerical calculations have been performed basing on the TBA equations of the spin-1/2 Fermi gas with attractive interaction (equation (19)). The TBA (equation (19)) involve infinite number of nonlinear integral equations accounting different lengths of spin strings (spin wave-bound states). This renders one to access the thermodynamics of the model analytically and numerically. The key observation is that for very large *n*, the function *a*_*n*_ (*x*)→0. For the string number *n* is greater than a critical cutoff value of the *n*_c_-length spin strings, the value 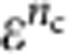 is independent of the interaction. Consequently, the contributions to the *ε*^u^ from higher spin strings, that is, *n* >*n*_c_, can be calculated analytically. By iteration, one finds that the value of *ε*^*n*^ for *n* >*n*_c_ is the same as the solution of the TBA (equation (26)) with *g*_1D_→∞; see ref. 35


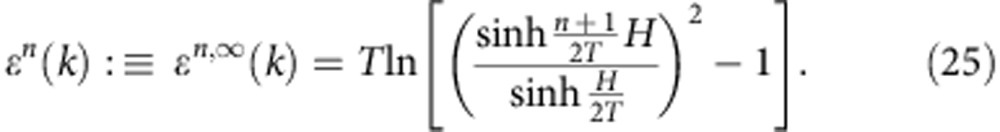


In our numerical programme, we fixed the value of *n*_c_ until the iteration error is small enough. In order to make a proper discretization in the variable space *k*, we need to find a cutoff *k*_c_ for the dressed energies *ε*^*n*^(*k*) in spin sector. For |*k*|→∞, we see *ε*^*n*^(*k*)→*ε*^*n*,∞^, which is given in equation (25), while for the charge sector *ε*^u,∞^=*ε*^b,∞^=∞. Therefore, for |*k*|>*k*_c_, we use this constant dressed energy *ε*^*n*,∞^ for numerical calculation. There exists an error in comparison with the real-dressed energies that is not flat in this region |*k*|>*k*_c_. In our programme, we also fix the value *kc* until the iteration error is negilible.

For an arbitrary interaction strength, we are able to truncate infinite number of strings TBA equations to finite number of TBA equations in terms of the variables *ε*^b,u^=*T* ln *ξ*^b,u^ and *ε*^*n*^=*T* ln*η*_*n*_


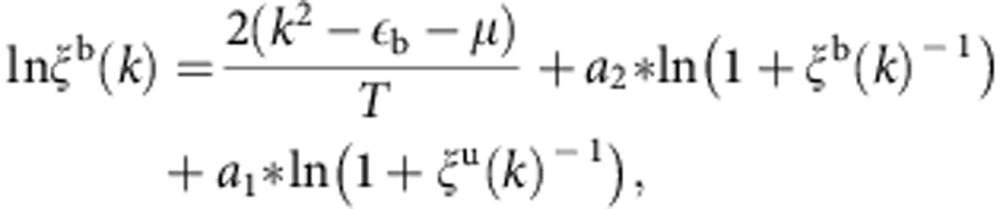



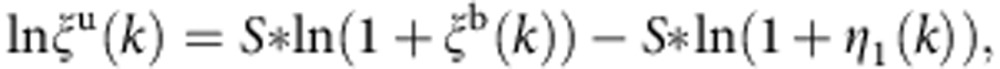



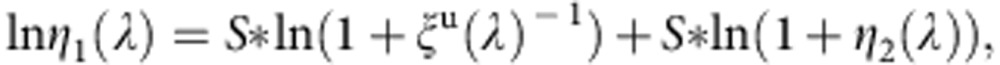



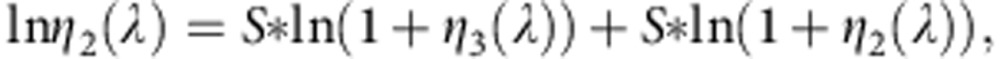


              …


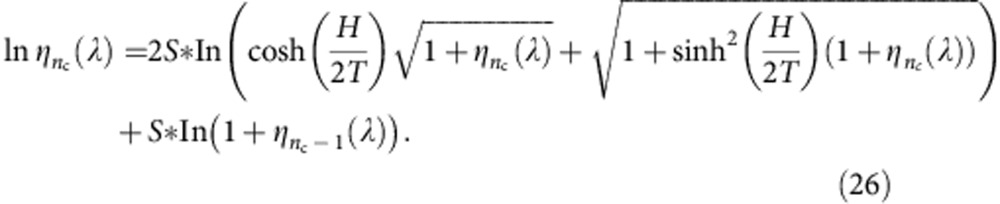


Here the functions 
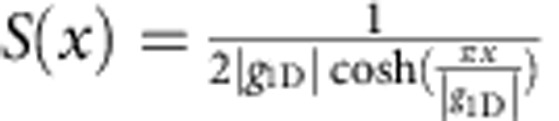
. From the parameters *ξ*^b^(*k*) and *ξ*^u^(*k*), we can get the pressures *p*^b,u^. This new set of the TBA equations provide numerical access to the full thermodynamics of the model, including the TLL physics, quantum criticality, thermodynamics and zero temperature phase diagram.

## Author contributions

Q.Z. and X.-W.G. conceived the project. Y.-Y.C. and Y.-Z.J. performed the numerical and analytical studies on the one-dimensional model. Q.Z and X.-W.G. wrote the paper.

## Additional information

**How to cite this article**: Chen, Y.-Y. *et al.* Critical behaviours of contact near phase transitions. *Nat. Commun.* 5:5140 doi: 10.1038/ncomms6140 (2014).

## Supplementary Material

Supplementary Information Supplementary Figures 1-4, Supplementary Note 1 and Supplementary References
