# Supplementary material for: Critical behaviours of contact near phase transitions
Source: Nat Commun. 2014 Oct 27;5:5140. doi: 10.1038/ncomms6140 (PMC4220487; doi:10.1038/ncomms6140)
Supplement: Supplementary Information — Supplementary Figures 1-4, Supplementary Note 1 and Supplementary References [file ncomms6140-s1.pdf]

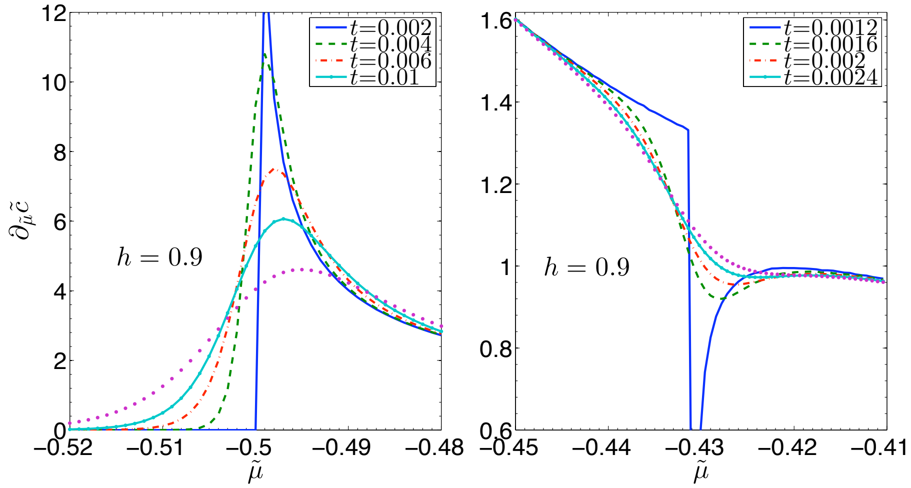

Supplementary Figure 1: Derivative of contact with respect to chemical potential vs $\tilde{\mu}$ near the phase transitions V-P (left panel) and P-PP (right panel) at different temperatures. Derivative of contact becomes divergent at $T = 0$ across these two critical points. At finite temperatures, such a divergence no longer exists. Here the critical chemical potentials $\tilde{\mu}_c = -0.5$ and $\tilde{\mu}_c = -0.431$ for V-P and P-PP phase transitions.

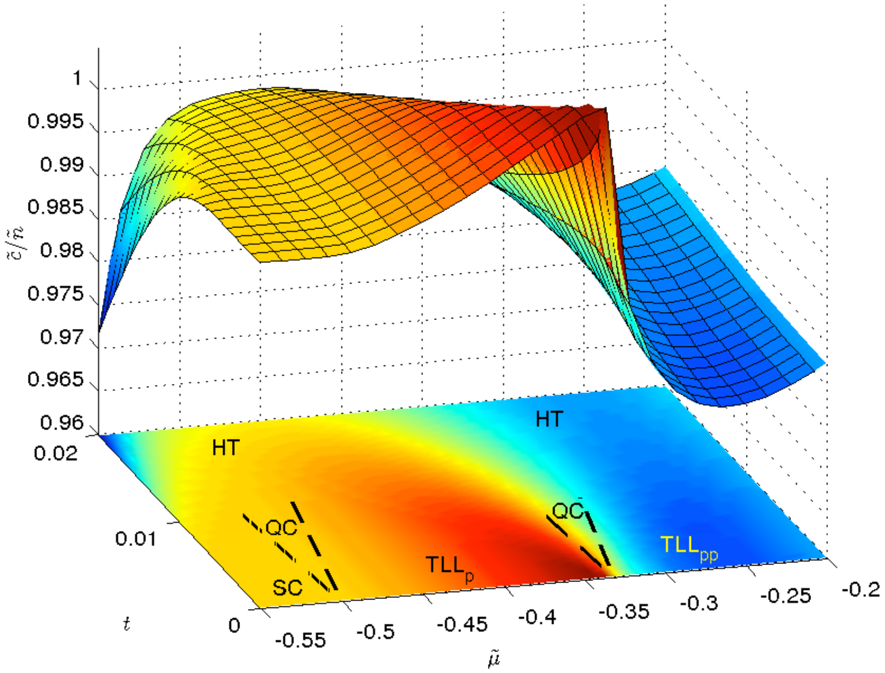

Supplementary Figure 2: A three-dimensional contour plot $\tilde{c}/\tilde{n}$ against t and $\tilde{\mu}$ at a fixed value of $h = 0.8$. Near two critical points $\tilde{\mu}_{c1} = -0.5$ and $\tilde{\mu}_{c2} = -0.335$, different scaling behaviour are visible. See the main text about the critical behaviors of contact.

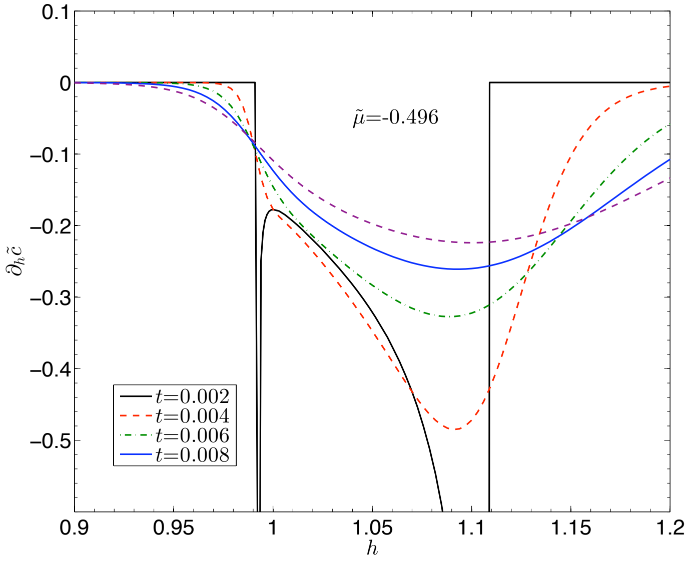

Supplementary Figure 3: Derivative of contact $\partial_h \tilde{c}$ vs h. Similar to $\partial_{\tilde{\mu}} \tilde{c}$, $\partial_h \tilde{c}$ also becomes divergent at $T = 0$ across the phase transition points. For a fixed value of $\tilde{\mu} = -0.496$ the first (second) divergent peak presents the critical behaviour of the gas for the phase transitions from P to PP and F to PP, respectively.

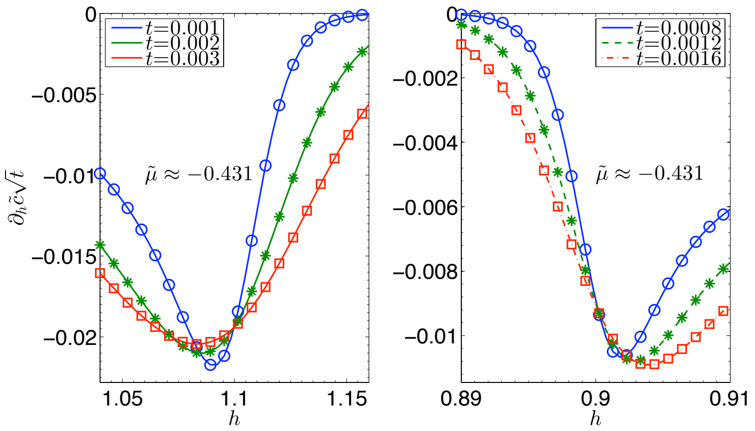

Supplementary Figure 4: Scaling behaviour of the derivative of contact $\partial_h \tilde{c} \sqrt{t}$ vs external field h. The left (right) panel shows the intersection of the derivatives of contact at different temperatures near the phase transition F-PP (P-PP). Here the critical field $h_c = 1.1$ and $h_c = 0.9$, respectively. This plot read off the critical dynamics exponent $z = 2$ and correlation length exponent $\nu = 1/2$ respectively.

Supplementary Note 1

Tan's contact

By definition of Tan's contact c

$$c = -\frac{g^2}{2} (\frac{\partial P}{\partial g})_{\mu,H,T} \tag{1}$$

and iterating the equations (20)-(23) in the main text we obtain

$$\begin{aligned}\widetilde{c} \;=\; & -\frac{1}{\sqrt{\pi}}t^{\frac{1}{2}}f_{1/2}^{A_{\rm b}}-\frac{1}{2\pi}t(f_{1/2}^{A_{\rm b}})^2-\frac{1}{\sqrt{2}\pi}tf_{1/2}^{A_{\rm u}}f_{1/2}^{A_{\rm b}}-\frac{1}{4\pi^{3/2}}t^{\frac{3}{2}}(f_{1/2}^{A_{\rm b}})^3\\ & -\frac{5}{2\sqrt{2}\pi^{3/2}}\left(f_{1/2}^{A_{\rm b}}\right)^2f_{1/2}^{A_{\rm u}}-\frac{1}{8\pi^2}t^2(f_{1/2}^{A_{\rm b}})^4-\frac{9}{4\sqrt{2}\pi^2}t^2\left(f_{1/2}^{A_{\rm b}}\right)^3f_{1/2}^{A_{\rm u}}\\ & -\frac{1}{\pi^2}t^2\left(f_{1/2}^{A_{\rm b}}\right)^2\left(f_{1/2}^{A_{\rm u}}\right)^2+\frac{7}{16\pi}t^2f_{1/2}^{A_{\rm b}}f_{3/2}^{A_{\rm b}}+\frac{1}{\sqrt{2}\pi}t^2f_{1/2}^{A_{\rm u}}f_{3/2}^{A_{\rm b}}\\ & +\frac{3}{\sqrt{2}\pi}t^2f_{1/2}^{A_{\rm b}}f_{3/2}^{A_{\rm u}}+O\left(t^{\frac{5}{2}}\right).\end{aligned}\tag{2}$$

Here we denote the dimensionless contact $\tilde{c}=c/\epsilon_{\rm b}^2$ and $f_n^x=Li_n(-e^{x/T})$. The above equation of contact looks very complex. Nevertheless, the universal scaling form of contact is hidden in such complexity of this kind. In the Supplementary Figure 2 shows a 3D contour plot $\tilde{c}/\tilde{n}$ against dimensionless temperature t and chemical potential $\tilde{\mu}$ at $h=0.8$. Near the lower critical point $\tilde{\mu}_{\rm c}=-0.5$, the flatness of $\tilde{c}/\tilde{n}$ is the consequence of the criticality of the model as discussed in the main paper. The values of $\tilde{c}/\tilde{n}$ drops very faster for the chemical potential excesses the upper critical point $\tilde{\mu}_{\rm c}=-0.335$ due to the increase of the polarization. We will present further discussions on the critical behaviour of contact in the following part.

The derivatives of contact connect various thermal and magnetic properties such as density, magnetization and entropy

$$\frac{1}{\epsilon_{\rm b}}\left(\frac{\partial c}{\partial\mu}\right)_{g,H,T} = -\left(\frac{\partial n}{\partial g}\right)_{\mu,H,T},\tag{3}$$

$$\frac{1}{\epsilon_{\rm b}}\left(\frac{\partial c}{\partial H}\right)_{g,\mu,T} = -\left(\frac{\partial m}{\partial g}\right)_{\mu,H,T},\tag{4}$$

$$\frac{1}{\epsilon_{\rm b}}\left(\frac{\partial c}{\partial T}\right)_{g,\mu,H} = -\left(\frac{\partial s}{\partial g}\right)_{\mu,H,T}.\tag{5}$$

We can analytically calculate these derivatives, namely

$$\begin{aligned}\partial_{\tilde{\mu}}\widetilde{c} \;=\; & -\frac{2}{\sqrt{\pi}}t^{-\frac{1}{2}}f_{-1/2}^{A_{\rm b}}-\frac{3}{\pi}f_{1/2}^{A_{\rm b}}f_{-1/2}^{A_{\rm b}}-\frac{2\sqrt{2}}{\pi}f_{1/2}^{A_{\rm u}}f_{-1/2}^{A_{\rm b}}-\frac{1}{\sqrt{2}\pi}f_{1/2}^{A_{\rm b}}f_{-1/2}^{A_{\rm u}}\\ & +t^{\frac{1}{2}}[-\frac{3}{\pi^{3/2}}(f_{1/2}^{A_{\rm b}})^2f_{-1/2}^{A_{\rm b}}-\frac{9\sqrt{2}}{\pi^{3/2}}f_{1/2}^{A_{\rm b}}f_{1/2}^{A_{\rm u}}f_{-1/2}^{A_{\rm b}}-\frac{1}{\pi^{3/2}}(f_{1/2}^{A_{\rm u}})^2f_{-1/2}^{A_{\rm b}}\\ & -\frac{9}{2\sqrt{2}\pi^{3/2}}(f_{1/2}^{A_{\rm b}})^2f_{-1/2}^{A_{\rm u}}]+\frac{1}{2}t[-\frac{5}{\pi^2}(f_{1/2}^{A_{\rm b}})^3f_{-1/2}^{A_{\rm b}}-\frac{30\sqrt{2}}{\pi^2}(f_{1/2}^{A_{\rm b}})^2f_{1/2}^{A_{\rm u}}f_{-1/2}^{A_{\rm b}}\\ & -\frac{27}{\pi^2}f_{1/2}^{A_{\rm b}}(f_{1/2}^{A_{\rm u}})^2f_{-1/2}^{A_{\rm b}}-\frac{9}{2\sqrt{2}\pi^2}(f_{1/2}^{A_{\rm b}})^3f_{-1/2}^{A_{\rm u}}-\frac{6\sqrt{2}}{\pi^2}(f_{1/2}^{A_{\rm b}})^2f_{-1/2}^{A_{\rm u}}\\ & -\frac{6}{\pi^2}(f_{1/2}^{A_{\rm b}})^2f_{1/2}^{A_{\rm u}}f_{-1/2}^{A_{\rm u}}+\frac{7}{4\pi}(f_{1/2}^{A_{\rm b}})^2+\frac{5\sqrt{2}}{\pi}f_{1/2}^{A_{\rm b}}f_{1/2}^{A_{\rm u}}+\frac{2}{\pi}f_{-1/2}^{A_{\rm b}}f_{3/2}^{A_{\rm b}}\\ & +\frac{\sqrt{2}}{\pi}f_{-1/2}^{A_{\rm u}}f_{3/2}^{A_{\rm b}}+\frac{8\sqrt{2}}{\pi}f_{-1/2}^{A_{\rm b}}f_{3/2}^{A_{\rm u}}]+O\left(t^{\frac{3}{2}}\right),\end{aligned}\tag{6}$$

$$\begin{aligned}\partial_h\widetilde{c} \;=\; & -\frac{1}{\sqrt{2}\pi}f_{1/2}^{A_{\rm u}}f_{-1/2}^{A_{\rm b}}-\frac{1}{2\sqrt{2}\pi}f_{1/2}^{A_{\rm b}}f_{-1/2}^{A_{\rm u}}+\frac{1}{\sqrt{2}}t^{\frac{1}{2}}[-\frac{3}{2\pi^{3/2}}f_{1/2}^{A_{\rm b}}f_{1/2}^{A_{\rm u}}f_{-1/2}^{A_{\rm b}}\\ & -\frac{1}{\sqrt{2}\pi^{3/2}}(f_{1/2}^{A_{\rm u}})^2f_{-1/2}^{A_{\rm b}}-\frac{5}{4\pi^{3/2}}(f_{1/2}^{A_{\rm b}})^2f_{-1/2}^{A_{\rm u}}]+\frac{1}{2}t[-\frac{3}{\sqrt{2}\pi^2}(f_{1/2}^{A_{\rm b}})^2f_{1/2}^{A_{\rm u}}f_{-1/2}^{A_{\rm b}}\\ & -\frac{15}{2\pi^2}f_{1/2}^{A_{\rm b}}(f_{1/2}^{A_{\rm u}})^2f_{-1/2}^{A_{\rm b}}-\frac{9}{4\sqrt{2}\pi^2}(f_{1/2}^{A_{\rm b}})^3f_{-1/2}^{A_{\rm u}}-\frac{3}{\pi^2}(f_{1/2}^{A_{\rm b}})^2f_{1/2}^{A_{\rm u}}f_{-1/2}^{A_{\rm u}}\\ & +\frac{3}{\sqrt{2}\pi}f_{1/2}^{A_{\rm b}}f_{1/2}^{A_{\rm u}}+\frac{1}{\sqrt{2}\pi}f_{-1/2}^{A_{\rm u}}f_{3/2}^{A_{\rm b}}+\frac{\sqrt{2}}{\pi}f_{-1/2}^{A_{\rm b}}f_{3/2}^{A_{\rm u}}]+O\left(t^{\frac{3}{2}}\right).\end{aligned}\tag{7}$$

Again, we can work out the scaling functions of these derivatives directly from the above equations. In Supplementary Figure 1, we plot the derivative of contact $\partial\tilde{c}/\partial\tilde{\mu}$ against chemical potential $\tilde{\mu}$. It is clearly see that the derivative of contact evolve into a sharp peak at the critical point.

Universal Scaling Forms

Quantum phase transitions occur at absolute zero temperature as the driving parameters μ and H are varied across the phase boundaries. The phase transitions are driven by quantum fluctuations with quantum critical points governed by divergent correlation lengths. Near a quantum critical point, the many-body system is expected to show universal scaling behaviour in the thermodynamic quantities. In the critical regime, a universal and scale-invariant description of the system is expected through the power-law scaling of the thermodynamic properties [3, 4]. Quantum phase transitions are uniquely characterized by the critical exponents depending only on the dimensionality and symmetry of the system. In order to work out the connection of Tan's contact to the criticality of the model, we first present the dimensionless functions

$$\tilde{A}_{\rm u} = A_{\rm u}/\epsilon_{\rm b} = \tilde{\mu} + h/2 + \frac{1}{\sqrt{\pi}} t^{\frac{3}{2}} f_{3/2}^{\tilde{A}_{\rm b}}, \tag{8}$$

$$\tilde{A}_{\rm b} = A_{\rm b}/\epsilon_{\rm b} = 2\tilde{\mu} + 1 + \frac{1}{2\sqrt{\pi}} t^{\frac{3}{2}} f_{3/2}^{\tilde{A}_{\rm b}} + \frac{\sqrt{2}}{\sqrt{\pi}} t^{\frac{3}{2}} f_{3/2}^{\tilde{A}_{\rm u}}. \tag{9}$$

From equations(8) and (9), we could expand contact (2) in the critical regime, i.e. $|\tilde{\mu} - \tilde{\mu}_{\rm c}| \ll 1$ and $|\tilde{\mu} - \tilde{\mu}_{\rm c}| > t$ near different quantum phase transitions.

V-P: From vacuum V to the fully-paired phase P, the critical point is $\tilde{\mu}_{\rm c} = -1/2, h < 1$. Taking low temperature limit near the critical point, we can obtain

$$\tilde{A}_{\rm u} \approx (\tilde{\mu} - \tilde{\mu}_{\rm c}) + (h-1)/2, \quad \tilde{A}_{\rm b} \approx 2(\tilde{\mu} - \tilde{\mu}_{\rm c}), \tag{10}$$

Substituting Eq.(10) into Eq.(2), we can obtain the scaling forms of contact and its derivative with respect to μ.

$$\widetilde{c} = -\frac{1}{\sqrt{\pi}} t^{\frac{1}{2}} Li_{\frac{1}{2}}(-e^{\frac{2(\tilde{\mu}-\tilde{\mu}_{\rm c})}{t}}), \tag{11}$$

$$\partial_{\tilde{\mu}} \widetilde{c} = -\frac{2}{\sqrt{\pi}} t^{-\frac{1}{2}} Li_{-\frac{1}{2}}(-e^{\frac{2(\tilde{\mu}-\tilde{\mu}_{\rm c})}{t}}).$$

In this phase $h_{\rm c}$ is the constant. Therefore there does not exist scaling form of the derivative respect to H, i.e. $\partial_h \widetilde{c} \approx 0$.

V-F: From the vacuum V to the fully-polarized phase F the critical point is $\tilde{\mu}_{\rm c} = -h/2, h > 1$. Near the critical point, we have obtain

$$\tilde{A}_{\rm u} \approx \tilde{\mu} - \tilde{\mu}_{\rm c}, \quad \tilde{A}_{\rm b} \approx 2(\tilde{\mu} - \tilde{\mu}_{\rm c}) + 1 - h \tag{12}$$

By expansion of Eq.(2) within the critical regime, the scaling form of contact is almost zero, i.e. $\widetilde{c} = -\frac{1}{\sqrt{\pi}} t^{\frac{1}{2}} Li_{\frac{1}{2}}(-e^{\frac{1-h}{t}}) \sim 0$. This regime does not exhibit universal scaling behaviour.

F-PP: From the fully-polaized phase F to the partially-polarized phase PP, the critical point is $\tilde{\mu}_{\rm c} = -1/2 + \frac{4}{3\pi}(h-1)^{3/2}$ and $h > 1$. Omitting the higher order contributions from t and $\tilde{\mu} - \tilde{\mu}_{\rm c}$ we can obtain

$$\tilde{A}_{\rm u} \approx (\tilde{\mu} - \tilde{\mu}_{\rm c}) + a/2, \quad \tilde{A}_{\rm b} \approx 2(\tilde{\mu} - \tilde{\mu}_{\rm c}) \tag{13}$$

where $a = (h-1)(1 + \frac{2}{3\pi}\sqrt{h-1})$. Substituting Eq.(13) into Eq.(2), we can get the scaling forms

$$\widetilde{c} = -\frac{1}{\sqrt{\pi}} t^{\frac{1}{2}} Li_{\frac{1}{2}}(-e^{\frac{2(\tilde{\mu}-\tilde{\mu}_{\rm c})}{t}})(1 - \frac{1}{\pi} a^{1/2} + \frac{1}{\pi} a^{3/2}), \tag{14}$$

$$\partial_{\tilde{\mu}} \widetilde{c} = t^{-\frac{1}{2}} Li_{-\frac{1}{2}}(-e^{\frac{2(\tilde{\mu}-\tilde{\mu}_{\rm c})}{t}})(-\frac{2}{\sqrt{\pi}} - \frac{4}{\pi^{3/2}} a^{1/2} + \frac{2}{\pi^{5/2}} a). \tag{15}$$

P-PP: Similar calculations can be carried out for the phase transitions from the phase P into phase PP, the critical point is $\tilde{\mu}_{\rm c} = -h/2 + \frac{4}{3\pi}(1-h)^{3/2}$ and $h < 1$. Thus near the critical pint, we have

$$\tilde{A}_{\rm u} \approx \tilde{\mu} - \tilde{\mu}_{\rm c}, \quad \tilde{A}_{\rm b} \approx 2(\tilde{\mu} - \tilde{\mu}_{\rm c}) + b, \tag{16}$$

where $b = (1-h)(1 + \frac{2}{\pi}\sqrt{1-h})$. Substituting Eq.(16) into Eq.(2), we obtain the scaling forms

$$\widetilde{c} = \tilde{c}_0 + t^{\frac{1}{2}} \lambda Li_{\frac{1}{2}}(-e^{\frac{\tilde{\mu}-\tilde{\mu}_{\rm c}}{t}}), \tag{17}$$

$$\partial_{\tilde{\mu}} \widetilde{c} = \tilde{c}_{d0} + t^{-\frac{1}{2}} \lambda_\mu Li_{-\frac{1}{2}}(-e^{\frac{\tilde{\mu}-\tilde{\mu}_{\rm c}}{t}}). \tag{18}$$

Where the constants are given by

$$\tilde{c}_0 = \frac{2}{\pi}b^{1/2} - \frac{2}{\pi^2}b + \frac{2}{\pi^3}b^{3/2},\ \lambda = \frac{\sqrt{2}}{\pi^{3/2}}b^{1/2} - \frac{5\sqrt{2}}{\pi^{5/2}}b + \frac{9\sqrt{2}}{\pi^{7/2}}b^{3/2} - \frac{2\sqrt{2}}{3\pi^{3/2}}b^{3/2},$$
$$c_{d0} = \frac{2}{\pi}b^{-1/2} + \frac{6}{\pi^2} + \frac{12}{\pi^3}b^{1/2},\ \lambda_\mu = \frac{\sqrt{2}}{\pi}b^{1/2} - \frac{9\sqrt{2}}{\pi^{5/2}}b.$$

In general, at quantum criticality, the above results can be cast into the universal scaling forms

$$\widetilde{c} = \widetilde{c}_0 + \lambda t^{(d/z)+1-(1/\nu z)}\mathcal{F}\left(\frac{\tilde{\mu}-\tilde{\mu}_c}{t^{1/\nu z}}\right), \tag{19}$$

$$\partial_{\tilde{\mu}}\widetilde{c} = \widetilde{c}_{d0} + \lambda_\mu t^{(d/z)+1-(2/\nu z)}\mathcal{G}\left(\frac{\tilde{\mu}-\tilde{\mu}_c}{t^{1/\nu z}}\right), \tag{20}$$

where the scaling functions read off the critical dynamic exponent $z = 2$, correlation exponent $\nu = 1/2$ for contact and its derivatives. In the above equations $\widetilde{c}_{0,d0}$, λ and λ_μ are constants. They are independent of the temperature. $\mathcal{F}(x)$, $\mathcal{G}(x)$ are universal dimensionless scaling functions. Despite the analytic results were derived for the strong atrractive case, the criticality is avaliable for all interaction strength. This nature is numerically confirmed in the main paper.

F-PP: From the phase F to the phase PP, the critical point is $h_c = 1 + 2(\tilde{\mu} + h/2)\left(1 - \frac{2\sqrt{2}}{3\pi}(\tilde{\mu}+h/2)^{\frac{1}{2}}\right)$. Near the critical point we have

$$\tilde{A}_u \approx (h - h_c)/2 + \alpha_1,\ \tilde{A}_b \approx \beta(h - h_c), \tag{21}$$

where $\alpha_1 = \left[\frac{3\sqrt{2}}{4}\pi(\tilde{\mu}+1/2)\right]^{\frac{2}{3}} - \frac{16}{3\sqrt{2}\pi}(\tilde{\mu}+1/2)^{\frac{3}{2}}$ and $\beta = \frac{1}{\sqrt{2}\pi}\left[3\sqrt{2}\pi(2\tilde{\mu}+1)\right]^{\frac{1}{3}}$. With the help of these function, we obtain

$$\partial_h\widetilde{c} = t^{-\frac{1}{2}}\,\mathrm{Li}_{-\frac{1}{2}}\left(-e^{\frac{\beta(h-h_c)}{t}}\right)\left(\frac{\sqrt{2}}{\pi^{3/2}}\alpha_1^{1/2} - \frac{2}{\pi^{5/2}}\alpha_1 - \frac{2\sqrt{2}}{3\pi^{3/2}}\alpha_1^{3/2}\right) \tag{22}$$

P-PP: From the phase P to the phase PP, the critical point is $h_c = -2\tilde{\mu} + \frac{16\sqrt{2}}{3\pi}(\tilde{\mu}+1/2)^{\frac{3}{2}}$. Near the critical point we have $\tilde{A}_u \approx (h-h_c)/2$, $\tilde{A}_b \approx \alpha$ where $\alpha = 2\tilde{\mu} + 1 - \frac{2}{3\pi}(2\tilde{\mu}+1)^{\frac{3}{2}}$. We obtain

$$\partial_h\widetilde{c} = t^{-\frac{1}{2}}\,\mathrm{Li}_{-\frac{1}{2}}\left(-e^{\frac{h-h_c}{2t}}\right)\left(\frac{1}{\sqrt{2}\pi^{\frac{3}{2}}}\alpha^{\frac{1}{2}} - \frac{5}{2\sqrt{2}\pi^{\frac{5}{2}}}\alpha - \frac{2\sqrt{2}}{3\pi^{\frac{3}{2}}}\alpha^{\frac{3}{2}} + \frac{9}{\sqrt{2}\pi^{\frac{7}{2}}}\alpha^{\frac{7}{2}}\right). \tag{23}$$

The above result of the scaling function in term of h can be also cast into the universal form

$$\partial_{\tilde{h}}\widetilde{c} = \widetilde{c}_{h0} + \lambda_h T^{(d/z)+1-(2/\nu z)}\mathcal{K}\left(\frac{h-h_c}{t^{1/\nu z}}\right). \tag{24}$$

Here $\widetilde{c}_{h0}$ and λ_h are constant.

Supplementary Figure 3 shows divergent behaviours of contact near the phase transitions P-PP and PP-F driven by the magnetic field h, see supplementary equation (14). Moreover, contact and its derivatives with respect to h at different temperatures must intersect at the critical point. This feature can be used to map out the phase boundaries from the trapped gas at nite temperatures. Supplementary Figure 4 shows the scaling behaviour described by Supplementary equations (29), (30) and (31).

Supplementary References

[1] Guan, X.-W. Batchelor, M. T., Lee, C. and Bortz, M., Phase transitions and pairing signature in strongly attractive Fermi atomic gases , Phys. Rev. B **76**, 085120 (2007).

[2] Guan, X.-W. and Ho, T.-L., Quantum Criticality of 1D Attractive Fermi Gas, Phys. Rev. A **84**, 023616 (2011).

[3] Fisher, M. P. A., Weichman, P. B. , Grinstein, G. and Fisher, D. S., Boson localization and the superfluid-insulator transition, Phys. Rev. B **40**, 546 (1989).

[4] Sachdev, S., *Quantum Phase Transitions*, Cambridge University Press (1999).

[5] Takahashi, M. , *Thermodynamics of One-Dimensional Solvable Models*, Cambridge University Press, Cambridge, (1999).
